# The Visit of North American Surgeons

**Published:** 1914-08-01

**Authors:** 


### THE VISIT OF NORTH AMERICAN SURGEONS.

The hospitals have certainly extended a warm welcome to the members of the Clinical Congress of North American Surgeons which has met in London during the week. Indeed, such an extensive programme was arranged that it may be wondered if our visitors may not have found themselves unable to see the wood for the trees. Even the plastic American brain must have found it a hard task to retain any adequate impression of more than a small proportion of the events presented to it for observation at this Conference; but doubtless most members satisfied themselves with attending just those operations which particularly concern their special interests in clinical surgery. If there is one thing more than another that tends to keep surgery alive it is the splendid competitive spirit that prevails amongst those whose life-work is devoted to the campaign against disease in our great voluntary institutions, where, unhampered by red tape, each individual surgeon is free to develop whatever line of research opens up before him—always, of course, having regard to the safety of the patients in his care. It has been interesting to note the growth of facilities for the mutual interchange of ideas that have been promoted in this country during recent years. Thus the surgical clubs which have been formed for the purpose of facilitating exchange of visits between the staffs of the leading hospitals have done much to increase the breadth of view of the workers at individual clinics.

Again, the systematic visits of American surgeons, initiated four or five years ago, to different centres in the United States and Canada, proved so helpful that they have now led to the assembly of an important and responsible Congress of North American operators in London, which is, after all, the most important centre of medical education in the world. Due recognition of the significance of the occasion has been given by the President of the Royal College of Surgeons, who consented to preside over the London Committee and opened the Congress. Further, the American Ambassador's welcoming address, and the fact that no less an authority than Professor von Eiselsberg, of Vienna, considered the occasion of sufficient moment to come over and open the debate on the operative treatment of gastric ulcer, demonstrate the importance of the Congress. American surgery has much to commend it at the present day, and more than one clinic on the North American continent has achieved world fame—notably that at Rochester, where the Mayo brothers have erected an extraordinary monument to their skill and strenuous efforts on behalf of scientific methods; having, indeed, founded an educational centre, a visit to which may soon be considered an essential part of the British surgeon's training. Nevertheless, whilst in boldness of technique and brilliance of imagination Transatlantic operations may lead the way, there can be no doubt that in the London operating theatres our visitors will find an accuracy of diagnosis, an attention to detail, and a mechanical thoroughness that are unsurpassed elsewhere. At the same time, it is very much to be hoped that the American delegates will have regarded the great London hospitals as something more than places where wonderful operations are performed; and that, amidst a multiplicity of engagements, the more astute of our visitors have found time to notice the high standard attained, both in administration and in the training of nurses, that characterises those institutions. Moreover, they may have possibly learnt a lesson from that spirit of voluntary service that is the keystone of our hospital system.

